# Notes and News

**Published:** 1904-02-20

**Authors:** 


### NOTES AND NEWS.

HOSPITAL MEETINGS.

Royal London Ophthalmic Hospital.—Annual Meeting at the Hospital on February 25th, at 3 P.M. Lord Avebury in the chair.

West End Hospital.—Annual Meeting at the Hospital on February 26th, at 4 P.M.

King's College Hospital.—Annual Meeting at the Hospital on February 25th, at 4 P.M.

THE Japanese ladies in England have combined in an endeavour to secure aid and sympathy for the families of Japanese soldiers and sailors who may be killed during the war.

THE Dowager Duchess of Russia has issued directions to the Russian Red Cross Society to make arrangements to provide for the care of the sick in the war. Very numerous offers of services have reached the society.

GLASGOW is to have a consumption sanatorium near Lanark Station. The Glasgow branch of the National Association for the Prevention of Tuberculosis has acquired a mansion suitable for adaptation, well situated at an elevation of about 600 feet above sea-level. The patients are to be accommodated in pavilions and chalets in the grounds. The pavilions will be erected by Messrs. Speirs, who are well known for the excellence of their structures of this kind. Each pavilion will contain five double-bedded rooms.

THE University of Cambridge announces an examination in tropical medicine and hygiene during 1904. The examination, lasting three days, will be held in August. Qualified medical men, of not less than twelve months' standing, are eligible for examination who can produce evidence of special study in the subject at a hospital for tropical diseases. The examination will be partly written, partly oral, practical and clinical. The clinical part will be conducted at a hospital for tropical diseases for purposes of diagnosis and comment. An extrance fee of six guineas is charged for the examination. Successful candidates are to receive a diploma in tropical medicine and hygiene. Applications for examination must be sent in by July 22nd, addressed to the Registrar of the University, Registry, Cambridge, where application forms are obtainable.

THE Childhood Society announces a course of public lectures to be given in the Library of the Sanitary Institute, 72 Margaret Street, during February and March.

THE expense to the public of an outbreak of smallpox is forcibly demonstrated by the experience of Foleshill in Warwickshire. Within the union there were 36 cases, and the expenses attending their treatment amounted to £602. The epidemics in this district last year were all traceable to navvies.

THE trustees of the late Mr. Primrose's estate have contributed £10,000 to the Aberdeen Royal Infirmary and £5,000 to the Sick Children's Hospital and the Morningside Hospital respectively. During the past year the trustees also made many useful gifts to the infirmary, amongst them being armchairs for the wards.

A CORRESPONDENCE appears in the *Times* between Lady Howe, Chairman of the Editing Committee of the Imperial Yeomanry Hospitals Report and Sir Alfred Fripp, in which he expresses regret at the strong criticisms passed by him on the statements made in the Report. Lady Howe on behalf of the Editing Committee accepts the explanation.

ON Monday and Tuesday last a fête was held at Claridge's Hotel in aid of the League of Mercy, under the direction of Lady Farquhar and a committee. The fête opened on Monday evening, and was held also on Tuesday afternoon. On both occasions the Grand Presidents, the Prince and Princess of Wales, honoured the proceedings by their presence; a most gracious act on their part, and a very real testimony to the interest they take in the Association and its work on behalf of the hospitals. The fête was attended by a very large number of influential people, and the entertainments included a *café chantant*, amateur theatricals, a display of fencing, of rapid sketching by Mr. Louis Wain, and a performance by Mr. Alfred de Rothschild's private band.

THE dinner given in honour of Mr. Timothy Holmes on February 13th by his colleagues, friends, and past pupils must have given great pleasure to the guest of the evening, the proceedings being marked with so much heartiness and appreciation. Mr. Timothy Holmes, who has now retired from the post of treasurer of St. George's Hospital, was joint author with Dr. Bristowe of a report on hospitals to the Privy Council which effected so many improvements in the administration of the London hospitals. The advances made since then have been so considerable that the reformer, once a Radical in the field of hospital administration, may now be regarded as a Tory. He has rendered immense service to medicine as a teacher. His system of surgery will survive as a classic amongst text-books. He has rendered equally valuable services in practical hospital administration by his occupation of the post of treasurer to the hospital where he was formerly consultant. By this act he placed at the disposal of the lay administration matured medical views and set an example that might be adopted with advantage by the consultants at other institutions.

